# Genetic variation and relationships of seven sturgeon species and ten interspecific hybrids

**DOI:** 10.1186/1297-9686-45-21

**Published:** 2013-06-28

**Authors:** Xiaomin Zhang, Wenhua Wu, Linmiao Li, Xufa Ma, Jinping Chen

**Affiliations:** 1 College of Fisheries, Huazhong Agricultural University, Wuhan, 430070, China; 2 Guangdong Entomological Institute/South China Institute of Endangered Animals, Guangzhou, 510260, China; 3 Heilongjiang River Fishery Research Institute (HRFRI) of Chinese Academy of Fishery Sciences, Harbin, 150070, China

## Abstract

**Background:**

Sturgeon cultivation is important for both industry and aquaculture in China. To date, more than 17 species or strains have been farmed for fillets and caviar production. Crossbreeding among different sturgeon species is frequent and the F2 hybrids are fertile. However, large-scale farming can have negative impacts on wild populations i.e. escape of exotic sturgeons and must be taken into consideration. Escape of exotic sturgeons can cause severe ecological problems, including threatening native sturgeon species once the exotic varieties become established or hybridize with native individuals. However, little is known about their genetic resources and variation.

**Methods:**

Genetic diversity and introgression of seven sturgeon species were analyzed using mitochondrial DNA *cytochrome oxidase subunit I* (*COI*) and nine microsatellite markers. This study included 189 individuals from seven sturgeon species and 277 individuals from ten lineages of F2 hybrid strains.

**Results:**

MtDNA *COI* sequences (632 bp long) were generated from 91 individuals across the 17 sturgeon strains and produced 23 different haplotypes. Haplotype diversity was high (h = 0.915 ± 0.015) and nucleotide diversity was low (π = 0.03680 ± 0.00153) in the seven sturgeon species and ten interspecific hybrids. Phylogenetic analyses resulted in almost identical tree topologies, and different haplotype structures were mainly related with sturgeons of different female parents. Analysis of molecular variance revealed that 81.73% of the genetic variance was due to matrilineal differences, while 9.40% resulted from strain variation. Pairwise Fst values obtained with POLYSAT software, were high among strains and ranged from 0.031 to 0.164. Admixture analysis assigned seven distinct groups and ten genotypes of admixed clusters composed of hybrid strains using STRUCTURE when assuming K = 7.

**Conclusions:**

The interspecific mtDNA gene tree corresponded to the expected taxonomic divisions. These relationships were also supported by the results from the microsatellite analysis and contributed to unambiguously identify seven sturgeon species and ten F2 hybrid strains from sturgeon farms in China. Moreover, we found that introgressive hybridization is pervasive, exists in both purebred and hybrid sturgeons, and may reflect widespread mismanagement in sturgeon breeding in China.

## Background

Hybridization is probably an inevitable process during speciation [1], in which offspring inherit restructured parental genes obtained by mating individuals of different genotypes. Hybridization is common both in plants and animals [2-4], and is largely exploited to improve various species [5-7], especially plants [8,9]. Through hybridization, desirable traits can be combined, leading to more competitive descendants and heterosis. Much research has been performed to evaluate crossbreeding in farm animals [10-12]. Crossbreeding is fast and effective, creates heterosis in the future generations [13,14] and can contribute to genetic improvement [15].

Sturgeon is one of the most ancient fish in the world and belongs to the order Acipenseriformes that contains 27 species divided into two families i.e. *Acipenseridae* (sturgeon, 25 species) and *Polydontidae* (paddlefish, two species). Most sturgeon species are near extinction [16], and have been listed in the Appendices to CITES (Convention on International Trade in Endangered Species of Wild Fauna and Flora) at the 10^th^ meeting of the Conference of the Parties (CoP10) since 1997. Today, sturgeons are considered worldwide as excellent candidates for aquaculture due to their high commercial value (caviar production and meat). Conflicting issues between the drastic reduction in natural populations and the huge profits in the business of sturgeon farming are the main driving force promoting the development of sturgeon aquaculture in China [17]. Recently, with the development of sturgeon aquaculture and the increase in cultured broodstock, China has become the largest sturgeon aquaculture country in the world, and hybrid sturgeons are widely bred [17]. The most recent survey has recorded more than 17 sturgeon strains (including both purebreds and hybrids) aquacultured in China, with two species i.e. *Acipenser baerii* and *A*. *schrenckii*, and three hybrid sturgeon strains i.e. *A*. *baerii* × *A*. *schrenckii*, *A*. *schrenckii* × *Huso dauricus*, and *A*. *baerii* × *A*. *gueldenstaedti* dominating sturgeon farming [17]. Among the hybrid sturgeon lines, some grow slowly and are of low quality value, but it is difficult to identify hybrid strains of sturgeon, especially at the early stages of growth i.e. fry and sub-adults. In addition, little is known on the genetics and polymorphism of sturgeons. In recent years, progress in artificial propagation technology has expedited the development of sturgeon aquaculture. However, when selective breeding is performed, the genetic relationships between candidate parents are unknown, resulting in random hybridization of different sturgeons. To prevent both inbreeding and degradation of germplasm resources, it is necessary to analyze the genetic background of the candidate breeders. Mitochondrial DNA (mtDNA) and microsatellite genetic markers have been used successfully in the studies of fish for introgressive hybridization [18-21]. In this study, we used nine microsatellites and the mitochondrial DNA (mtDNA) gene *cytochrome oxidase subunit I (COI)* to examine the genetic diversity, introgression, and differences in genetic background of 17 farmed sturgeon strains, including seven species and ten hybrids.

## Methods

### Animals and DNA extraction

From 2008 to 2011, seven sturgeon species and ten hybrids were obtained from the Engineering and Technology Center of Sturgeon Breeding and Cultivation of Chinese Academy of Fishery Science (Beijing, China), the Heilongjiang Fisheries Research Institute, the Beijing Fisheries Research Institute, and the Hangzhou Qiandaohu Xunlong Sci-Tech Co., Ltd. (Table 1). The historical background of all the sturgeons collected was known, and each species was identified according to anatomical characteristics. Fish fins were stored in 95% ethanol. Genomic DNA was prepared using a DNeasy tissue kit (Qiagen).

**Table 1:** **Sturgeon species and code**, **number of individuals and line type used in the experiment**

**Species**	**Species code**	**Number of individuals**	**Type**
*A*. *schrenckii*	S	30	Purebred
*A*. *baerii*	X	30	Purebred
*A*. *gueldenstaedti*	E	34	Purebred
*H*. *dauricus*	H	26	Purebred
*A*. *ruthenus*	Xi	23	Purebred
*A*. *sinensis*	Z	25	Purebred
*A*. *stellatus*	G	21	Purebred
*A*. *baerii*♀ × *H*. *dauricus*♂	XH	31	Hybrid
*A*. *baerii*♀ × *A*. *schrenckii*♂	XS	32	Hybrid
*A*. *schrenckii*♀ × *A*. *baerii*♂	SX	30	Hybrid
*A*. *baerii*♀ × *A*. *gueldenstaedti*♂	XE	23	Hybrid
*A*. *gueldenstaedti*♀ × *A*. *baerii*♂	EX	40	Hybrid
*A*. *gueldenstaedti*♀ × *H*. *dauricus*♂	EH	30	Hybrid
*H*. *dauricus*♀ × *A*. *schrenckii*♂	HS	30	Hybrid
*A*. *schrenckii*♀ × *H*. *dauricus*♂	SH	26	Hybrid
*A*. *ruthenus*♀ × *H*. *dauricus*♂	XiH	30	Hybrid
*A*. *sinensis*♀ × *A*. *schrenckii*♂	ZS	5	Hybrid

### Mitochondrial DNA gene *COI* amplification and microsatellite genotyping

The mtDNA gene *COI* (632 bp) was amplified for five to eight individuals of each sturgeon strain (total n = 91) using published primers that were designed specifically for fish i.e. (FishF1 [5′-TCAACCAACCACAAAGACATTGGCAC-3′] and FishR1 [5′-TAGACTTCTGGG TGGCCA AAGAATCA-3′]) [22]. PCR (polymerase chain reaction) amplifications were carried out in 25 μL reaction volumes containing approximately 20 ng of template DNA, 1 μL of each primer, 9.5 μL of ddH_2_O, and 12.5 μL of 2x Premix Taq DNA polymerase (Takara). Amplification conditions were as follows: 95°C for 2 min; 35 cycles at 94°C for 30 s, 57°C for 30 s, and 72°C for 1 min; and a final extension at 72°C for 10 min. Positive (with DNA template) and negative (with water) controls were used to check PCR performance and contamination. The PCR products were purified using the PCR purification kit (Shanghai Bio-Tec, Ltd) and sequenced with the ABI PRISM BigDye Terminator Ready Reaction kit (Applied Biosystems) and run on an ABI 377 genetic analyzer according to the manufacturer’s protocol. To avoid errors in sequencing, PCR amplifications of all samples were sequenced on both strands.

Fifteen microsatellite loci were selected before the start of the experiment, the amplified products were separated by electrophoresis on 10% (29/1 Acrylamide-bisacrylamide) 0.50 mm thick denaturing polyacrylamide gels (120 V, 10 h), and DNA bands were visualized using silver staining. The size of individual alleles was determined using a 50 bp DNA size standard (Tiangen) and allele sizes were estimated by Gelpro 3.2. Nine of 15 most effective loci were selected for further analyses after the preliminary analysis [See Additional file 1: Table S1] [23,24]. The corresponding nine primer pairs were fluorescently labeled with one of the following dyes FAM, HEX, or TAMRA. PCR was performed under the following conditions: preheating at 94°C for 4 min; denaturing at 94°C for 40 s, annealing at 50-58°C for 30 s, and elongation at 72°C for 40 s for 35 cycles; extension at 72°C for 10 min. Amplifications were carried out in 15 μL reaction volumes containing 10 ng of template DNA, 0.5 μL of each primer, 6 μL of ddH_2_O, and 7.5 μL of 2x Premix Taq DNA polymerase (Takara). All PCR were carried out in a PTC-100 thermal cycler (MJ Research). Allele sizing was carried out by automated fluorescent scanning detection in an ABI 377XL DNA sequencer (Applied Biosystems) using ROX500 as internal lane size standard, and the software GENESCAN and GENOTYPER (Applied Biosystems).

### Sequence analysis

Nucleotide sequences were aligned using the CLUSTALX software (1.83) and checked by eye. Variable nucleotide sites (including transversions (tv), transitions (ts) and insertions/deletions) were calculated using MEGA 2.1 software. Haplotypes were estimated using DNASP, and the haplotype network was drawn using NETWORK software. For the phylogenetic analysis, Modeltest 3.06 was run to determine the appropriate model of molecular evolution in a likelihood ratio test framework. We then performed maximum-parsimony (MP) analysis using the programs PAUP 4.0 and MRBAYES 3.0, with *Polypterus bichir bichir* as out-group. Gaps were treated as missing in the parsimony analyses. Bootstrap analyses were performed with 6000 replicates and 1000 full heuristic replicates for maximum parsimony. For Bayesian phylogenetic inference, four Markov chain Monte Carlo (MCMC) simulations were run for 1 500 000 generations, sampling every 1000 generations. The initial 5% of trees were discarded as burn-in, and finally, a 50% majority rule consensus tree was constructed.

Specific nucleotide sites were analyzed in the *COI* sequences, and haplotype distributions, nucleotide diversities (π) within and among strains were estimated using DNASP. Hierarchical analysis of molecular variance (AMOVA) was performed to compare the levels of genetic diversity within and among several possible strain groups using Arlequin 3.11 with 1023 permutations.

### Microsatellite analysis

Individual polyploidy genotypes were scored from microsatellite banding patterns in the electropherograms according to the Microsatellite DNA Allele Counting-Peak Ratios (MAC-PR) method of Esselink et al.(2004) [25]. The STRUCTURE version 2.3.2 program [26] was used to infer strain composition based on microsatellite data. We used the Bayesian clustering approach in STRUCTURE 2.0 to identify the most likely number of clusters (K) as well as to assign individuals to these clusters. We performed five replicate runs (burn-in period of 1 000 000 steps and 120 000 MCMC iterations) at each value of K from 2 to 7. Individuals were assigned on the basis of their membership coefficient and factor correlation analysis (FCA) based on the multilocus genotypes was carried out using the GENETIX program to separate the strains and identify any intermediate genotypes resulting from admixture of strains [27].

We used the POLYSAT software to calculate the number of alleles, allele frequency, and Shannon-Wiener index. The Simpson index was used to characterize the levels of genetic diversity in each sturgeon strain [28].

## Results

### Mitochondrial DNA

MtDNA *COI* sequences (632 bp) were generated from 17 strains represented by 91 individuals. One hundred and six variable nucleotide sites were found, including 14 transversions (tv), 82 transitions (ts) and 10 insertions/deletions, and 23 haplotypes were defined [GenBank accession numbers: KC578823-KC578845]. Our results revealed high haplotype diversity (h = 0.915 ± 0.015) and low nucleotide diversity (π = 0.03680 ± 0.00153) in the seven sturgeon species and ten interspecific hybrids. The seven pure sturgeon species harbored unique nucleotide sites: *A*. *ruthenus* (Xi) had the greatest number of specific nucleotide sites (i.e. 14), while *A*. *baerii* (X) and *A*. *gueldenstaedti* (E) had the lowest number (i.e. 1) [See Additional file 2: Table S2]. H09 was the most common haplotype shared by 19 individuals and *A*. *ruthenus*♀ × *H*. *dauricus*♂ (XiH) had the biggest number of haplotypes (Table 2).

**Table 2:** Summary of mtDNA COI region haplotype distribution among the 17 sturgeon species

**Species**	**XH**	**X**	**XS**	**SX**	**S**	**SH**	**XE**	**EX**	**E**	**EH**	**H**	**H**	**XiH**	**Xi**	**ZS**	**Z**	**G**	**Total**
**Haplotype**																		
H01	1																	1
H02	2	2																4
H03	2	3	5															10
H04				5														5
H05					1													1
H06					1													1
H07					1													1
H08					2													2
H09							6	4	5	4								19
H10								1										1
H11										1								1
H12											1							1
H13											4	4						8
H14												1						1
H15						5							1					6
H16													5					5
H17													1					1
H18													1					1
H19														5				5
H20															5	5		10
H21																1		1
H22																	4	4
H23																	2	2
Total	5	5	5	5	5	5	6	5	5	5	5	5	8	5	5	6	6	91

Code for species is presented in Table 1.

**Figure 1:**
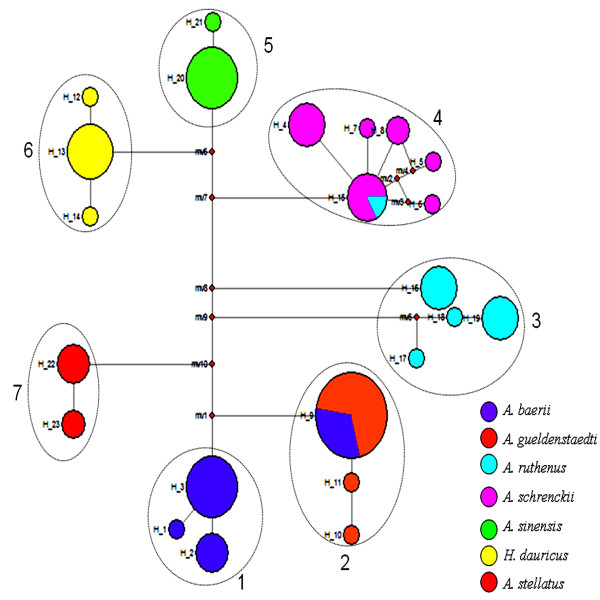
**Statistical parsimony network based on the mtDNA gene *COI *haplotypes.** Each circle represents a single haplotype; circle size is scaled by haplotype frequency; the same colors indicate the same matrilineal sturgeon; 1: *A*. *baerii*, *A*. *baerii*♀ × *H*. *dauricus*♂ and *A*. *baerii*♀ × *A*. *schrenckii*♂; 2: *A*. *gueldenstaedti*, *A*. *gueldenstaedti*♀ × *A*. *baerii*♂, *A*. *gueldenstaedti*♀ × *H*. *dauricus*♂ and *A*. *baerii*♀ × *A*. *gueldenstaedti*♂; 3: *A*. *ruthenus* and *A*. *ruthenus*♀ × *H*. *dauricus*♂; 4: *A*. *schrenckii*, *A*. *schrenckii*♀ × *A*. *baerii*♂, *A*. *schrenckii*♀ × *H*. *dauricus*♂ and *A*. *ruthenus*♀ × *H*. *dauricus*♂; 5: *A*. *sinensis* and *A*. *sinensis*♀ × *A*. *schrenckii*♂; 6: *H*. *dauricus* and *H*. *dauricus*♀ × *A*. *schrenckii*♂; 7: *A*. *stellatus*.

Using MODELTEST, the HKY+G model was built using the best fitting distance estimator. It showed a gamma distribution shape parameter of 0.2655, a transition/transversion (Ti/Tv) ratio of 4.0901, and base frequencies of A = 0.2650, C = 0.2825, G = 0.1780 and T = 0.2744. Parsimony analyses were performed under equal weight (Ti/Vi = 1) and unequal weight (Ti/Vi = 4.0901) sets. All phylogenetic analyses resulted in almost identical tree topologies. With the exception of all individuals from *A*. *baerii*♀ × *A*. *gueldenstaedti*♂ (XE) and one individual from *A*. *ruthenus*♀ × *H*. *dauricus*♂ (XiH), most of the tree topologies were grouped together with the same matrilineal sturgeon [See Additional file 3: Figure S1]. This observation was supported by haplotype network analysis (Figure 1).

AMOVA analysis revealed that 81.73% of the genetic variance was due to matrilineal differences and 9.40% to strain variation, while only 8.86% of the genetic variance originated from strains within groups with the same matrilineal sturgeon (Table 3).

**Table 3:** **AMOVA results for sturgeon *COI *gene estimated using F**-**statistics**

**Source of variation**	**d**.**f**.	**Sum of squares**	**Variance components**	**% of variation**	**F**-**statistics (*P***-**value)**
Among groups	6	910.209	11.26227	81.73	FST:0.90597* (*P* = 0.00000)
Among populations within groups	10	76.851	1.22116	8.86	FSC:0.48521*(*P* = 0.00293)
Within populations	74	95.875	1.29561	9.40	FCT:0.81735* (*P* = 0.00000)
Total	90	1082.934	13.77904		

* *P* < 0.01; d.f. = degree of freedom.

### Microsatellites

A total of 466 individual sturgeons (Table 1) were genotyped using nine nuclear microsatellite markers [See Additional file 4: Table S3]. All tested microsatellite loci were polymorphic in all farmed sturgeons. Microsatellite LS68 had the biggest number of alleles per individual, while LS19 had the smallest. *A*. *sinensis*♀ × *A*. *schrenckii*♂ (ZS) tended to have more alleles per individual per locus, while *A*. *baerii*♀ × *H*. *dauricus*♂(XH) had the fewest [See Additional file 5: Table S4].

**Figure 2:**
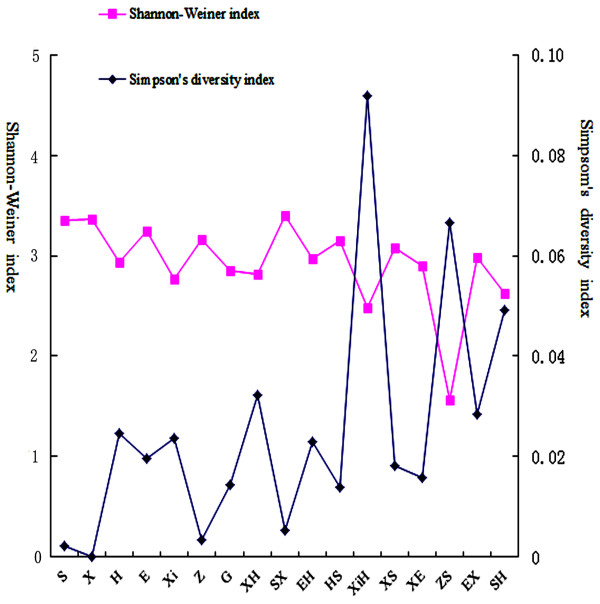
**The Shannon**-**Wiener and the Simpson indices of 17 sturgeon strains.**

Using POLYSAT software, we showed that the total number of alleles observed at each locus ranged from 7 to 15, with a total of 386 alleles detected across the nine loci. The total number of alleles found within strains ranged from 42 (*A*. *sinensis*♀ × *A*. *schrenckii*♂) to 117 (*H*. *dauricus*♀ × *A*. *schrenckii*♂), and the number of alleles per microsatellite ranged from 25 (LS54) to 71 (SPL106) [See Additional file 4: Table S3]. Shannon-Wiener and Simpson indices were used as measures of allelic diversity and dominance in the population, respectively. Based on the microsatellite analysis, the Shannon-Wiener and Simpson indices of sturgeon alleles ranged from 1.561 to 3.404 and from 0 to 0.092, respectively (Figure 2).

**Figure 3:**
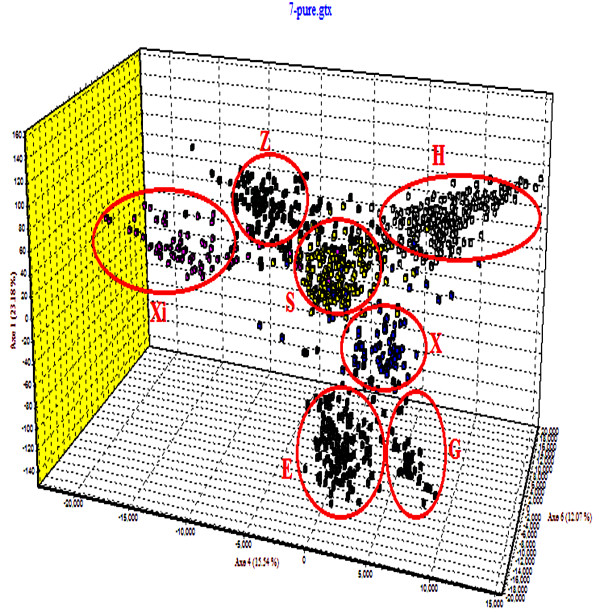
**Factorial Correspondence Analysis** (**FCA**) **based on nine microsatellite loci in seven purebred sturgeon species.**

Factor correlation analysis (FCA) based on microsatellite genotypes revealed a clear segregation among purebred sturgeon species and hybrids. FCA highlighted the differences among the seven analyzed purebred species. The most informative is axis 1 (23.18% of the total genetic variation), which separates the seven species and distinguishes *A*. *gueldenstaedti* (E) and *A*. *baerii* (X) from the five other species. Axis 2, which is slightly less informative (15.54%), separates *A*. *ruthenus* (Xi), *A*. *sinensis* (Z), *A*. *schrenckii* (S) and *A*. *stellatus* (G). Finally, axis 3 mainly separates *A*. *gueldenstaedti* (E) and *H*. *dauricus* (H) with 12.07% of the total genetic variation (Figure 3). The ten hybrids, even with their typical mtDNA *COI* haplotypes of female descent, are identified as hybrids, but occupy an intermediate position among the parents according to the multidimensional analysis of microsatellite genotyping data [See Additional file 6: Figure S2, Additional file 7: Figure S4, Additional file 8: Figure S5, Additional file 9: Figure S6, Additional file 10: Figure S7, Additional file 11: Figure S8 and Additional file 12: Figure S9).

To investigate the existence of different genetic clusters in sturgeon breeds, the admixture model implemented by the STRUCTURE software was used to analyze separately the microsatellite genotyping data. The assignment test, which was conducted with STRUCTURE with K ranging from 2 to 7 and was based on nine microsatellites, separated the samples into seven distinct groups, congruent with the seven sturgeon species, and ten mixed-cluster groups representing the ten interspecific hybrid strains [See Additional file 13: Figure S3]. Various degrees of introgressive hybridization were also observed in all seven purebred sturgeons with *H*. *dauricus* showing the highest level.

## Discussion

### Molecular identification

DNA barcoding is widely used for species determination because sequence divergences are generally much lower among individuals of a given species than among closely related species [29-32]. However, hybridization among species can cause taxonomic uncertainty. Since mtDNA is maternally inherited, any hybrid or subsequent generation will only have maternal mtDNA [31].

Jenneckens et al. (2000) reported that genetic contamination of *A*. *gueldenstaedti* with *A*. *baerii* or *A*. *baerii* hybrids occurred in the Volga River in Russia. Crosses and backcrosses of these species with native *A*. *gueldenstaedti* led to the loss of the morphological diagnostic *A*. *baerii* features [33]. In our study, we also used nine microsatellite loci from nuclear DNA and mtDNA COI for sturgeon identification. Our data showed that mtDNA can identify purebred sturgeon species and the maternal origin of hybrids (Figure 1) and [See Additional file 3: Figure S1] since each line harbors unique sites [See Additional file 2: Table S2]. The interspecific mtDNA gene tree constructed agreed with the expected taxonomic divisions, except for all individuals of *A*. *baerii*♀ × *A*. *gueldenstaedti*♂ (XE) and one individual of *A*. *ruthenus*♀ × *H*. *dauricus*♂ (XiH). *A*. *baerii*♀ × *A*. *gueldenstaedti*♂ (XE) was grouped together with strains bred from the female parent *A*. *gueldenstaedti*, perhaps because of sex-specific or nonreciprocal crossing. Moreover, *A*. *baerii* may be a hybrid and may have been backcrossed many times with *A*. *gueldenstaedti*. Hybridization may be involved in particular gender combinations in each species due to the maternal inheritance of mtDNA [34]. One individual of *A*. *ruthenus*♀ × *H*. *dauricus*♂ (XiH), identified within the wrong cluster, may be due to erroneous identification of the original specimen.

In general, matrocliny and patrocliny with different combinations of dominant and recessive alleles of the genes determine the diagnostic characters. These clearly demonstrate how the hybrid individuals acquire varying degrees of similarity and differences from the parental species [35]. Taxonomic confusion as a result of interspecific hybridization does not seem to be a major issue and can be solved with genetic markers of matroclinous and patroclinous inheritance [36]. Mitochondrial DNA sequences are frequently transferred to the nucleus, giving rise to the so-called nuclear mtDNA sequences or NUMT (nuclear mitochondrial DNA) because of hybridization [37]. NUMT is not helpful for species identification because of its potential pseudogene status [31]. NUMT are common in plants and animals, but few NUMT are found in fish [38,39]. In our results, since no evidence of significant linkage disequilibrium (LD) was found among any loci, we can avoid this negative factor of NUMT for random distribution of these loci. Microsatellite results can distinguish reciprocal crosses among different sturgeons [See Additional file 6: Figure S2, Additional file 7: Figure S4, Additional file 8: Figure S5, Additional file 9: Figure S6, Additional file 10: Figure S7, Additional file 11: Figure S8 and Additional file 12: Figure S9), and a few samples were assigned to the ‘wrong’ congeneric species, which may represent introgressive hybridization.

### Introgression

Microsatellite analysis reveals population structure and helps to explain past introgression of DNA among sturgeon species. Strong signatures are widely documented when comparing introgression of microsatellites with that of mtDNA [36,40]. We found that introgressive hybridization is pervasive in either purebred or hybrid farmed sturgeons, which may reflect widespread mismanagement of sturgeon breeding in China. STRUCTURE results showed that introgressive hybridization in domestic varieties (*A*. *schrenckii*, *H*. *dauricus*, and *A*. *sinensis*) is less serious than in varieties introduced to China from abroad (*A*. *baerii*, *A*. *gueldenstaedti*, *A*. *ruthenus*, and *A*. *stellatus*). *H*. *dauricus* is the most obvious example of strain impurity with a high level of genetic information originating from another sturgeon [See Additional file 13: Figure S3], which may be due to the fact that this species was frequently used as a parent in crosses. Our results also showed that *A*. *stellatus* was almost assimilated by *A*. *baerii* and that *A*. *ruthenus* had introgressed into the hybrid stains of *A*. *baerii* as a parent, especially in *A*. *baerii*♀ × *A*. *schrenckii*♂ and *A*. *gueldenstaedti*♀ × *A*. *baerii*♂. Such sturgeon hybridization has also been reported between the endangered *A*. *ruthenus* species and the exotic *A*. *baerii* species in the Danube River and between *A*. *baerii* and *A*. *ruthenus* in the Irtysh River of Sinkiang [41,42]. Campton reported that introgression can cause a more abundant species to genetically assimilate a rare species [43].

In China, it is easy for farmed sturgeons to escape to natural water systems where wild sturgeons live, and there are no behavioral or physiological barriers preventing interbreeding between the cultured and wild populations [44]. *A*. *sinensis* is distributed in the Yangtze River Basin, but recently, different types of sturgeons have been cultured along the tributaries, and escaped individuals have crossed with *A*. *sinensis*, leading to its farraginous genotype.

### Genetic diversity

MtDNA results revealed high gene diversity and low nucleotide diversity in these farmed sturgeons. Results from AMOVA analysis for the 17 strains of sturgeon showed that most of the genetic variance was distributed among the different matrilineal strains. These conclusions were supported by our microsatellite results. Since Shannon-Wiener and Simpson indices measure allelic gene diversity and dominance in the population, respectively, a high and homogeneous Shannon-Wiener index may indicate that hybrid regions could comprise a large number of multigenerational backcrossed hybrids that are indistinguishable from the parental species [45], and that multigenerational backcrossed hybrids resemble the parental species with which they share most of their alleles [46]. *A*. *ruthenus*♀ × *H*. *dauricus*♂ (XiH) showed the highest Simpson index, which may be due to the fact that introgression of a few loci may promote adaptive divergence [1]. Similarly, high levels of genetic diversity have been reported in lake sturgeons across the species’ range [46]. The low levels of genetic diversity in *A*. *sinensis*♀ × *A*. *schrenckii*♂ (ZS) suggest that *A*. *sinensis*♀ × *A*. *schrenckii*♂ (ZS) was founded by a small number of individuals.

Genetic diversity of farm animal is often exploited to meet current production needs, to allow sustained genetic improvement, and to facilitate rapid adaptation to changing breeding objectives [47]. Allele introgression is crucial to livestock genetics, while crossbreeding has always been a staple of breeding programs. Hybridization and polyploid formation will continue to generate species diversity . Our results show that farmed sturgeons hybridize with each other to varying degrees and with high genetic diversity. The conservation aquaculture programs are designed to minimize the genetic impacts on wild populations caused by introgressive farmed populations.

## Conclusions

We combined a morphological study with DNA barcoding and microsatellite markers to show that we can unambiguously identify seven sturgeon species and ten F2 hybrid strains from sturgeon farms in China. We found that introgressive hybridization is pervasive and exists in both purebred and hybrid farmed sturgeons, which may reflect widespread mismanagement of sturgeon breeding in China. We also found that farmed sturgeons hybridize with each other to varying degrees and with high genetic diversity.

## Competing interests

The authors declare that they have no competing interests.

## Authors’ contributions

XMZ and WHW contributed equally to this work. XMZ performed data analysis and wrote the manuscript. WHW conceived the original ideas. LML and XFM helped to perform the analysis and improved the manuscript. JPC conceived the study, made substantial contribution to the interpretation of the results, and revised the manuscript. All authors read and approved the manuscript.

## Supplementary Material

Additional file 1: Table S1 Characteristics of sturgeon microsatellite markers in this study. The data provided represent characteristics of the nine most effective microsatellite loci which were selected for analyses in this work. Click here for file

Additional file 2: Table S2 Specific nucleotide sites observed in the mtDNA COI sequences of seven purebred sturgeon species. The data provided represent specific nucleotide sites analyzed by MEGA, all the sites were observed in mtDNA COI sequences of seven purebred sturgeon species. Click here for file

Additional file 3: Figure S1 Phylogenetic tree obtained by Bayesian inference from the analysis of COI sequences with *Polypterus bichir bichir* as out-group. The figure represents the phylogenetic tree infered from the analysis of COI sequences with *Polypterus bichir bichir* as out-group. Different colors represent different matrilineal. Click here for file

Additional file 4: Table S3 Total number of alleles detected for the nine microsatellite loci selected for the analysis of 17 sturgeon strains. The data provides the number of alleles detected for the nine microsatellite loci by POLYSAT. Click here for file

Additional file 5: Table S4 Average number of alleles per individual per locus detected in 17 sturgeons. The data provides the average number of alleles per individual per locus detected in 17 sturgeon strains. N is the total number of individuals for each sturgeon strain. Click here for file

Additional file 6: Figure S2 Factorial Correspondence Analysis (FCA) based on nine microsatellite loci in *A*. *baerii*, *A*. *gueldenstaedti* and their hybrids. The figure shows the distinction between *A*. *baerii*, *A*. *gueldenstaedti* and *A*. *baerii*♀ × *A*. *gueldenstaedti*♂ and *A*. *gueldenstaedti*♀ × *A*. *baerii*♂. Click here for file

Additional file 7: Figure S4 Factorial Correspondence Analysis (FCA) based on nine microsatellite loci in *A*. *schrenckii*, *H*. *dauricus* and their hybrids. The figure shows the distinction between *A*. *schrenckii*, *H*. *dauricus* and *A*. *schrenckii*♀ × *H*. *dauricus*♂ and *H*. *dauricus*♀ × *A*. *schrenckii*♂. Click here for file

Additional file 8: Figure S5 Factorial Correspondence Analysis (FCA) based on nine microsatellite loci in *A*. *baerii*, *A*. *schrenckii* and their hybrids. The figure shows the distinction between *A*. *baerii*, *A*. *schrenckii* and *A*. *baerii*♀ × *A*. *schrenckii*♂ and *A*. *schrenckii*♀ × *A*. *baerii*♂. Click here for file

Additional file 9: Figure S6 Factorial Correspondence Analysis (FCA) based on nine microsatellite loci in *A*. *schrenckii*, *A*. *sinensis* and their hybrids. The figure shows the distinction between *A*. *schrenckii*, *A*. *sinensis* and *A*. *sinensis*♀ × *A*. *schrenckii*♂. Click here for file

Additional file 10: Figure S7 Factorial Correspondence Analysis (FCA) based on nine microsatellite loci in *H*. *dauricus*, *A*. *ruthenus* and their hybrids. The figure shows the distinction between *H*. *dauricus*, *A*. *ruthenus* and *A*. *ruthenus*♀ × *H*. *dauricus*♂. Click here for file

Additional file 11: Figure S8 Factorial Correspondence Analysis (FCA) based on nine microsatellite loci in *A*. *baerii*, *H*. *Dauricus* and their hybrids. The figure shows the distinction between *A*. *baerii*, *H*. *dauricus* and *A*. *baerii*♀ × *H*. *dauricus*♂. Click here for file

Additional file 12: Figure S9 Factorial Correspondence Analysis (FCA) based on nine microsatellite loci in *A*. *gueldenstaedti*, *H*. *dauricus* and their hybrids. The figure shows the distinction between *A*. *gueldenstaedti*, *H*. *dauricus* and *A*. *gueldenstaedti*♀ × *H*. *dauricus*♂. Click here for file

Additional file 13: Figure S3 Assignation of the 466 sturgeons by STRUCTURCE analysis based on nine microsatellite loci in 17 sturgeon strains. The figure illustrates the existence of different genetic clusters in sturgeon breeds revealed the by the analysis of microsatellite genotyping data. Click here for file
