# Supplementary material for: Genetic variation and relationships of seven sturgeon species and ten interspecific hybrids
Source: Genet Sel Evol. 2013 Jun 28;45(1):21. doi: 10.1186/1297-9686-45-21 (PMC3704922; doi:10.1186/1297-9686-45-21)
Supplement: Additional file 1: Table S1 — Characteristics of sturgeon microsatellite markers in this study. The data provided represent characteristics of the nine most effective microsatellite loci which were selected for analyses in this work. [file 1297-9686-45-21-S1.doc]

Table S1 Characteristics of sturgeon microsatellite markers in this study

| Locus | Primer sequences(5’-3’) | Core  sequences | Fluorescent  dye | Tm  (℃) |
| --- | --- | --- | --- | --- |
| SPL106 | F:CACGTGGATGCGAGAAATAC  R:GGGGAGAAAACTGGGGTAAA | (CTAT)_12_ | 5’HEX | 58 |
| SPL113 | F: TCCCACATGGCTTGTATTGA  R: ACCACACCATGCGTCATAAG | (AGAT)_14_ | 5’TAMRA | 58 |
| SPL120 | F: ATTCCATGAGCAACACCACA  R: TGATGGTCTGATGAGATCGG | (TATC)_15_ | 5’HEX | 58 |
| SPL168 | F: CACTGATTCGCTACAACCGT  R: AGAAGGACTTGCAGTCCGAA | (TATC)_18_ | 5’HEX | 50 |
| LS19 | F: CATCTTAGCCGTCTGTGGTAC  R: CAGGTCCCTAATACAATGGC | (TTG)_9_ | 5’TAMRA | 58 |
| LS54 | F: CTCTAGTCTTTGTTGATTACAG  R: CAAAGGACTTGAAACTAGG | (GATA)_6_  (GACA)_7_ | 5’TAMRA | 56 |
| LS68 | F: TTATTGCATGGTGTAGCTAAAC  R: AGCCCAACACAGACAATATC | (GATA)_13_ | 5’TAMRA | 57 |
| AS100 | F: GGGAGAAAACTGGGGTAAA  R:CCAAAAGAAGAATGGTAGACGG | (AAC)_2_  (TAAA)_9_ | 5’HEX | 57 |
| HLJSX24 | F: TGTCTCTGCGTGTGTGTGAA  R: CTGCAGCTCCATCTTCTCCT | (CT)_6N_(CT)_6N_  (CT)_20_ | 5’6FAM | 58 |
