# Supplementary material for: Genetic variation and relationships of seven sturgeon species and ten interspecific hybrids
Source: Genet Sel Evol. 2013 Jun 28;45(1):21. doi: 10.1186/1297-9686-45-21 (PMC3704922; doi:10.1186/1297-9686-45-21)
Supplement: Additional file 2: Table S2 — Specific nucleotide sites observed in the mtDNA COI sequences of seven purebred sturgeon species. The data provided represent specific nucleotide sites analyzed by MEGA, all the sites were observed in mtDNA COI sequences of seven purebred sturgeon species. [file 1297-9686-45-21-S2.doc]

|  | 68 | 110 | 125 | 131 | 161 | 164 | 173 | 188 | 200 | 203 | 224 | 248 | 249 | 257 | 269 | 287 | 296 | 299 | 318 | 320 | 326 | 329 | 374 | 380 | 386 | 401 | 407 | 419 | 431 | 440 | 446 | 452 | 458 | 459 | 461 | 482 | 485 | 491 | 501 | 506 | 509 | 518 | 539 | 548 | 578 | 605 | 608 | N |
| --- | --- | --- | --- | --- | --- | --- | --- | --- | --- | --- | --- | --- | --- | --- | --- | --- | --- | --- | --- | --- | --- | --- | --- | --- | --- | --- | --- | --- | --- | --- | --- | --- | --- | --- | --- | --- | --- | --- | --- | --- | --- | --- | --- | --- | --- | --- | --- | --- |
| X | A | T | A | C | C | C | T | G | C | G | C | T | T | C | G | G | T | C | C | G | T | A | G | G | T | T | T | T | C | A | A | G | T | C | A | C | G | A | A | A | G | A | C | C | A | C | A | 1 |
| S | G | C | - | - | - | - | - | - | - | - | - | - | - | T | - | - | - | - | - | - | - | - | - | - | - | - | - | C | - | - | G | - | - | - | - | - | - | - | - | - | - | - | - | - | G | - | - | 5 |
| E | G | - | - | - | - | - | - | - | - | - | - | - | - | - | - | - | - | - | - | - | - | - | - | - | - | - | - | - | - | - | - | A | - | - | - | - | - | - | - | - | - | - | - | - | - | - | - | 1 |
| H | G | - | ­­- | - | - | - | - | - | - | A | - | C | - | - | - | - | - | - | - | A | - | G | - | - | - | - | - | - | - | C | - | - | - | - | - | T | - | - | - | - | - | - | - | - | - | - | - | 7 |
| Xi | G | - | - | - | G | - | - | - | - | - | - | - | - | - | A | A | C | T | T | - | C | - | - | C | - | - | - | - | T | - | - | - | C | - | G | - | A | - | T | G | A | - | - | - | - | - | - | 14 |
| Z | G | - | - | - | - | - | - | - | T | - | - | - | C | - | - | - | - | - | - | - | - | - | - | - | - | - | C | - | - | - | - | - | - | - | - | - | - | G | - | - | - | G | T | T | - | - | - | 7 |
| G | G | - | G | T | - | T | C | A | - | - | T | - | - | - | - | - | - | - | - | - | - | - | A | - | C | C | - | - | - | - | - | - | - | T | - | - | - | - | - | - | - | - | - | - | - | T | G | 12 |

Table S2 Specific nucleotide sites observed at mtDNA COI sequences among different seven purebred sturgeon species
