# Supplementary figures and images for: Genetic variation and relationships of seven sturgeon species and ten interspecific hybrids
Source: Genet Sel Evol. 2013 Jun 28;45(1):21. doi: 10.1186/1297-9686-45-21 (PMC3704922; doi:10.1186/1297-9686-45-21)

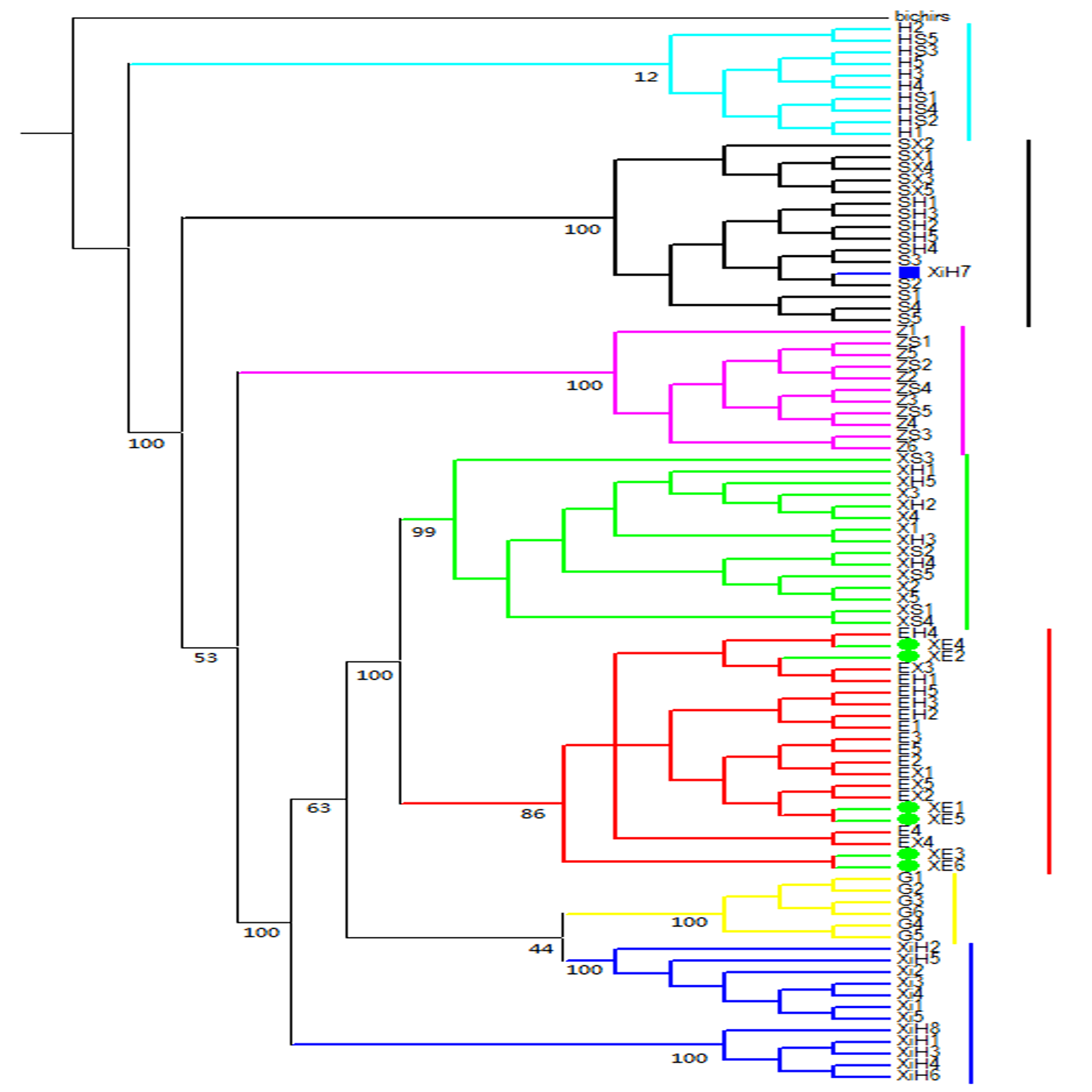

Supplement: Additional file 3: Figure S1 — Phylogenetic tree obtained by Bayesian inference from the analysis of COI sequences with Polypterus bichir bichir as out-group. The figure represents the phylogenetic tree infered from the analysis of COI sequences with Polypterus bichir bichir as out-group. Different colors represent different matrilineal. [file 1297-9686-45-21-S3.png]

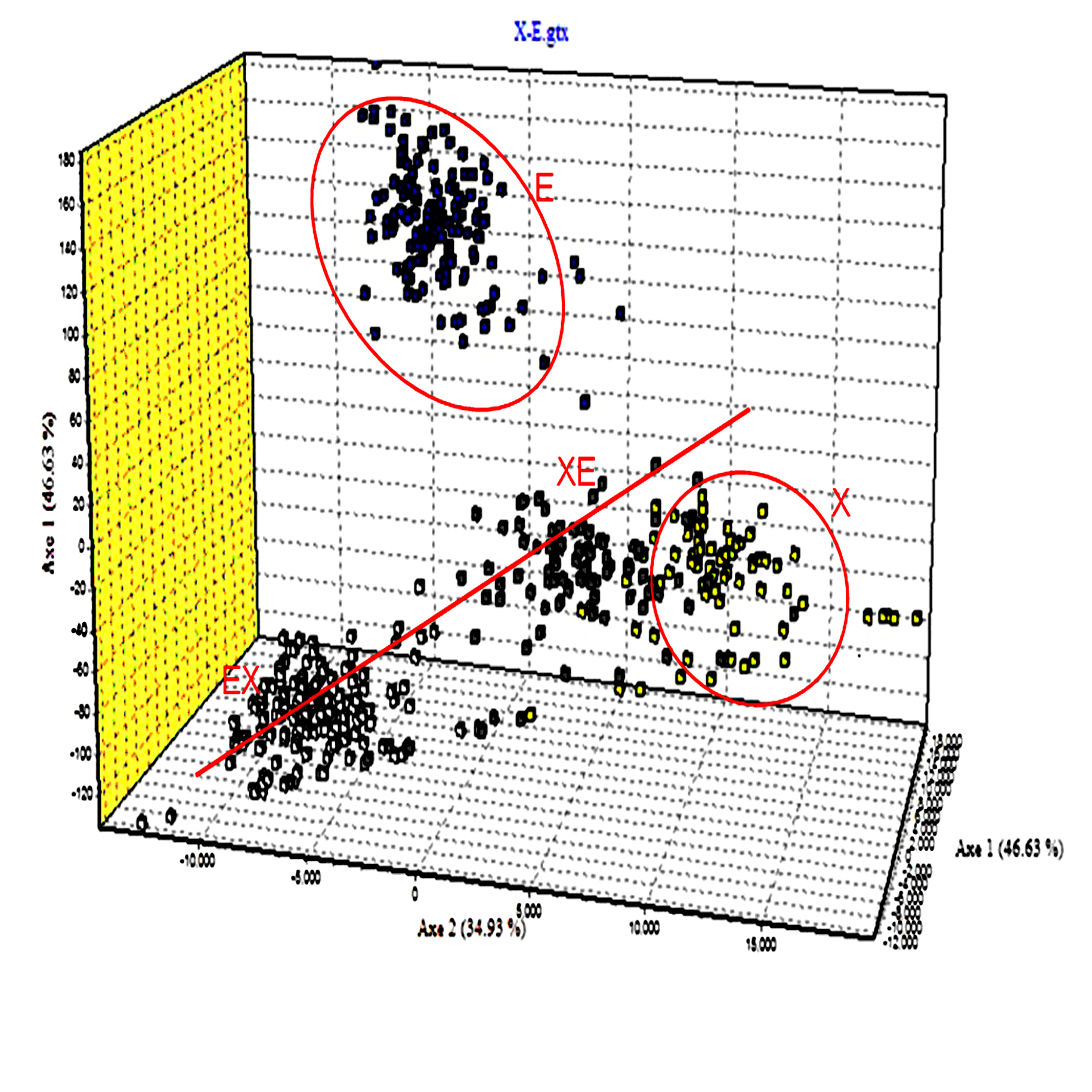

Supplement: Additional file 6: Figure S2 — Factorial Correspondence Analysis (FCA) based on nine microsatellite loci in A. baerii, A. gueldenstaedti and their hybrids. The figure shows the distinction between A. baerii, A. gueldenstaedti and A. baerii♀ × A. gueldenstaedti♂ and A. gueldenstaedti♀ × A. baerii♂. [file 1297-9686-45-21-S6.png]

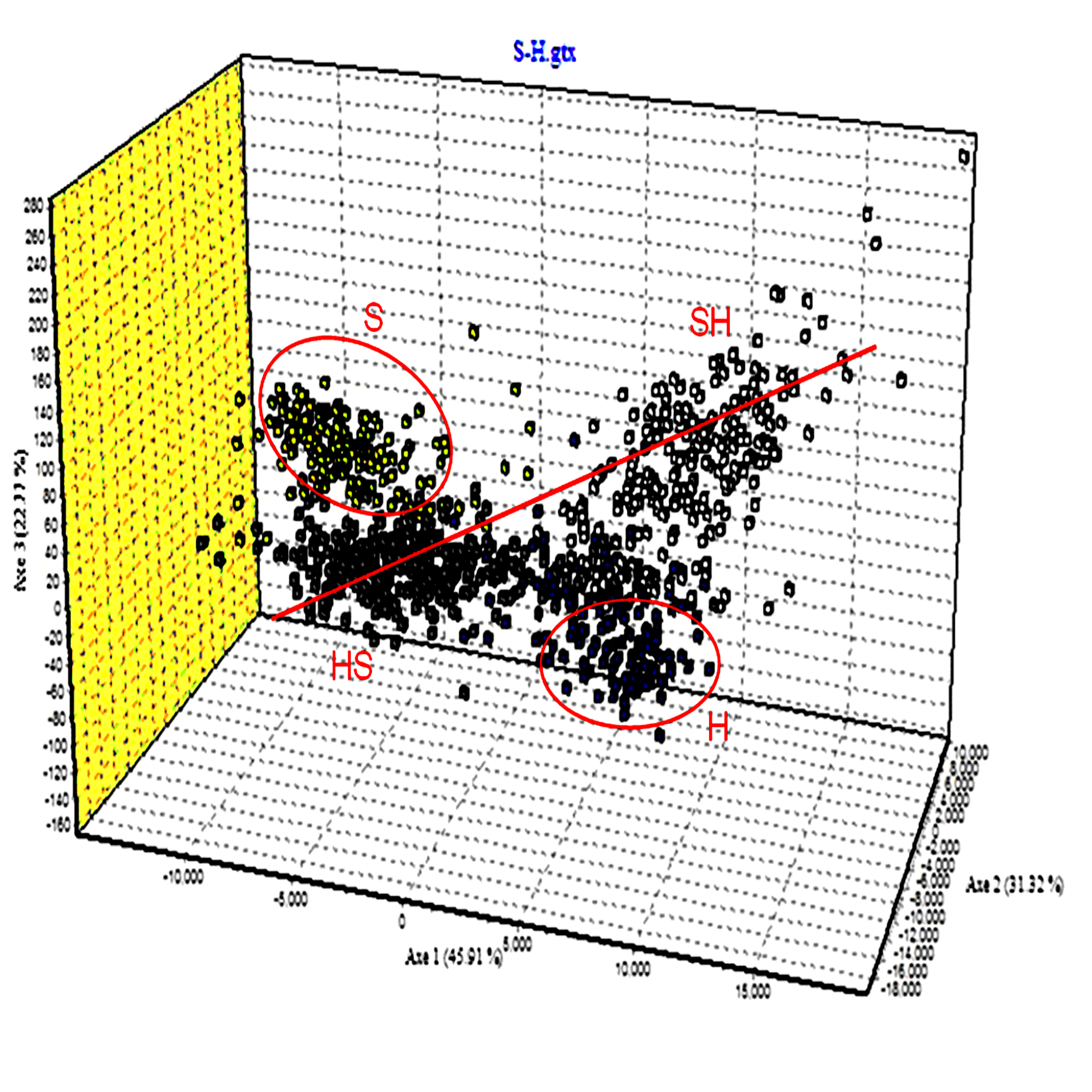

Supplement: Additional file 7: Figure S4 — Factorial Correspondence Analysis (FCA) based on nine microsatellite loci in A. schrenckii, H. dauricus and their hybrids. The figure shows the distinction between A. schrenckii, H. dauricus and A. schrenckii♀ × H. dauricus♂ and H. dauricus♀ × A. schrenckii♂. [file 1297-9686-45-21-S7.png]

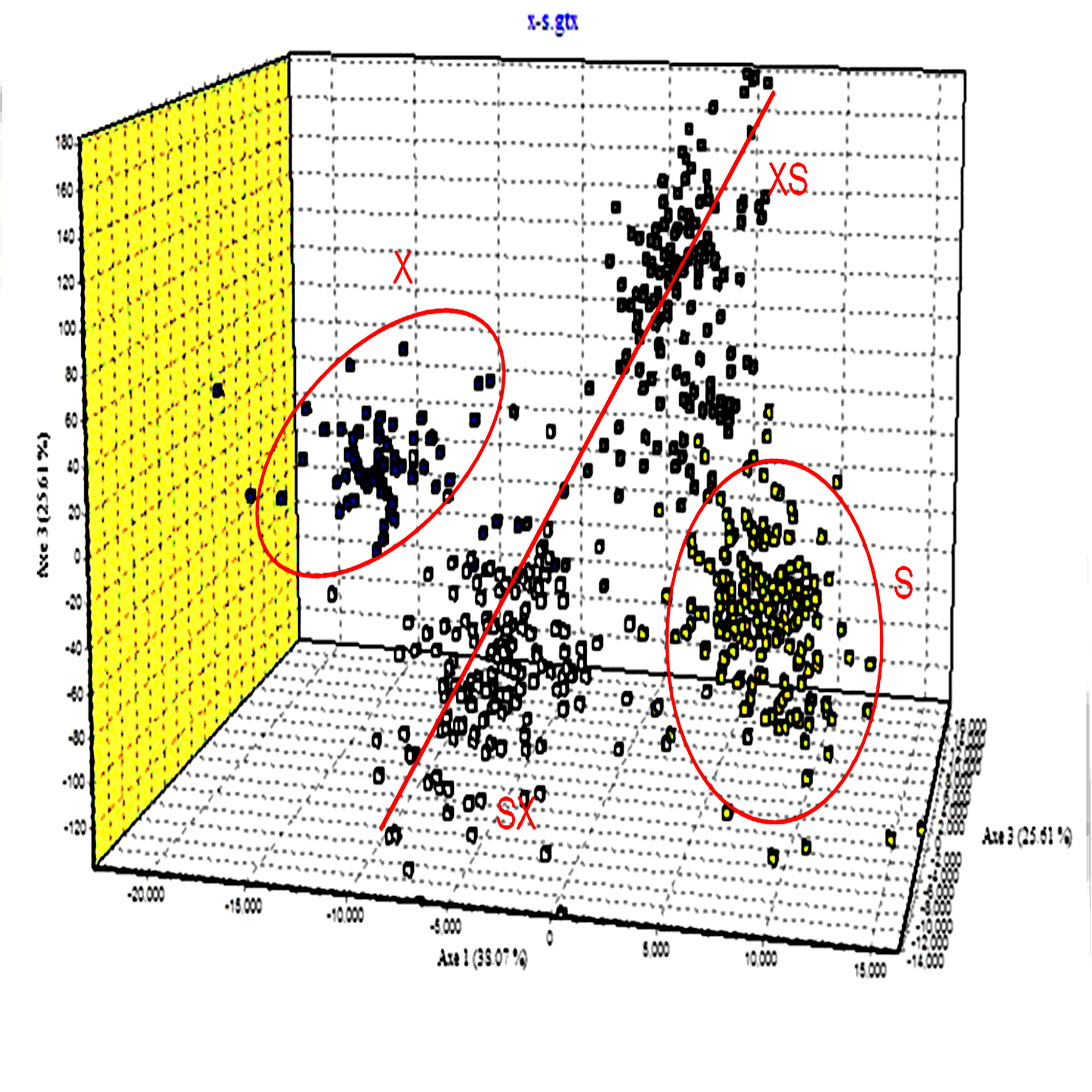

Supplement: Additional file 8: Figure S5 — Factorial Correspondence Analysis (FCA) based on nine microsatellite loci in A. baerii, A. schrenckii and their hybrids. The figure shows the distinction between A. baerii, A. schrenckii and A. baerii♀ × A. schrenckii♂ and A. schrenckii♀ × A. baerii♂. [file 1297-9686-45-21-S8.png]

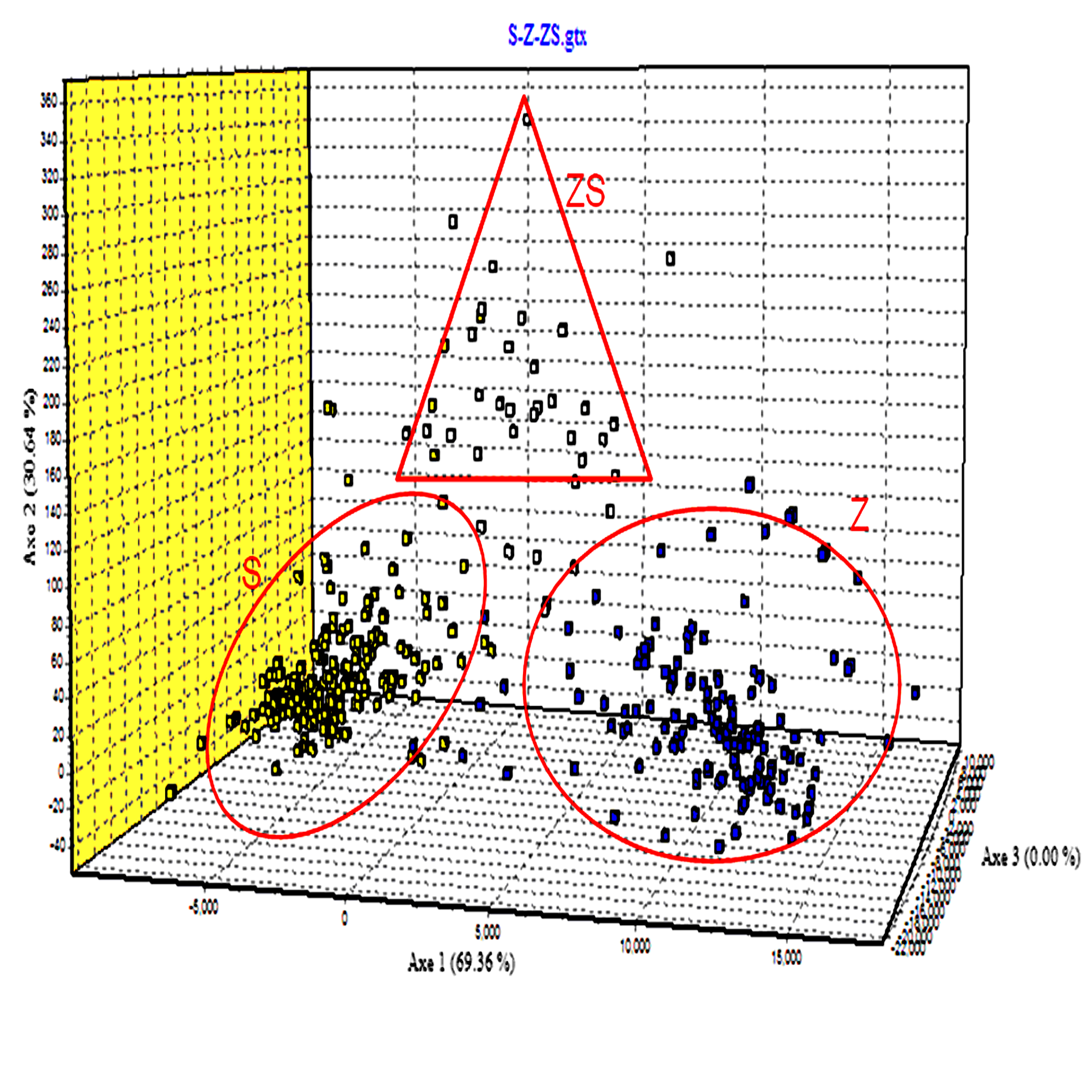

Supplement: Additional file 9: Figure S6 — Factorial Correspondence Analysis (FCA) based on nine microsatellite loci in A. schrenckii, A. sinensis and their hybrids. The figure shows the distinction between A. schrenckii, A. sinensis and A. sinensis♀ × A. schrenckii♂. [file 1297-9686-45-21-S9.png]

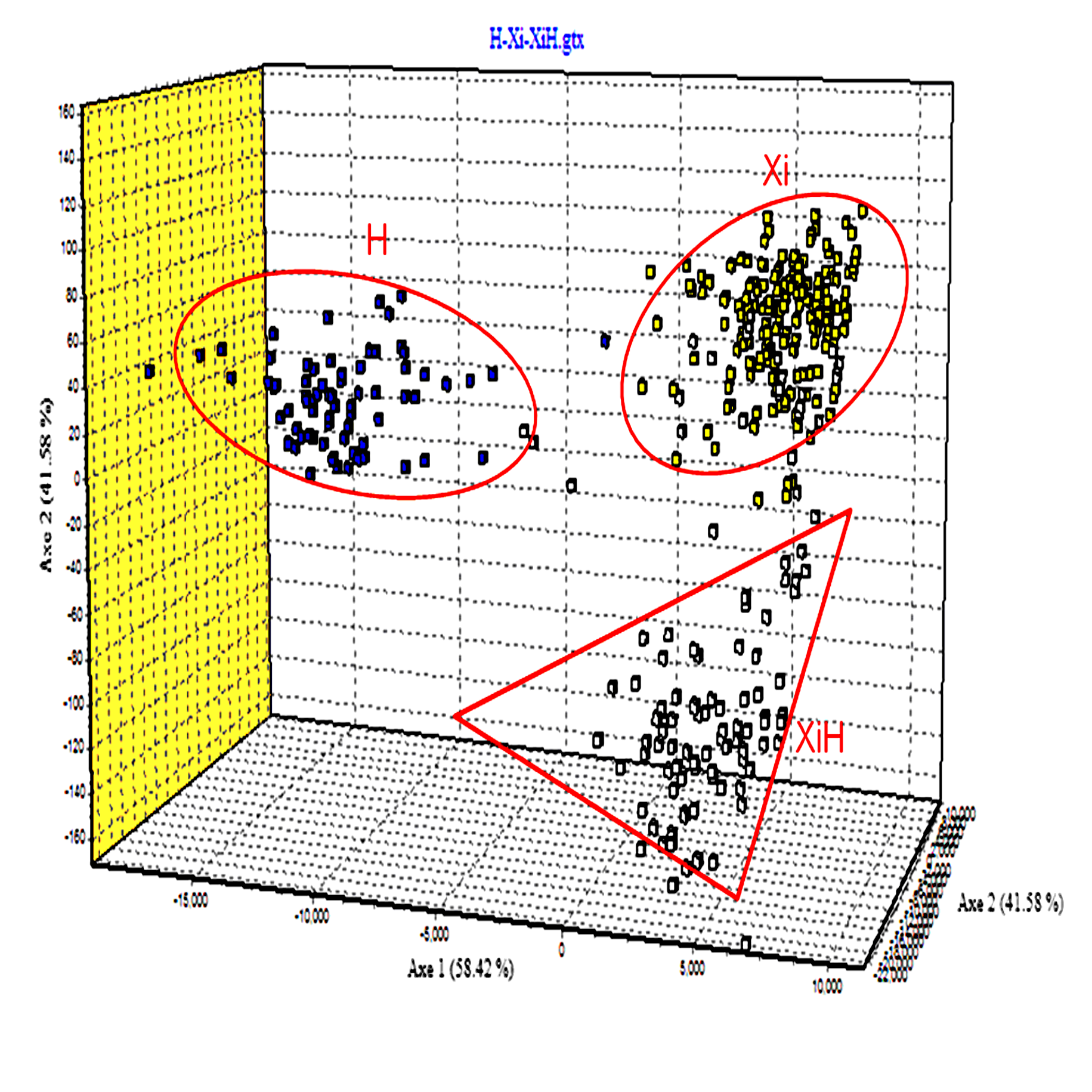

Supplement: Additional file 10: Figure S7 — Factorial Correspondence Analysis (FCA) based on nine microsatellite loci in H. dauricus, A. ruthenus and their hybrids. The figure shows the distinction between H. dauricus, A. ruthenus and A. ruthenus♀ × H. dauricus♂. [file 1297-9686-45-21-S10.png]

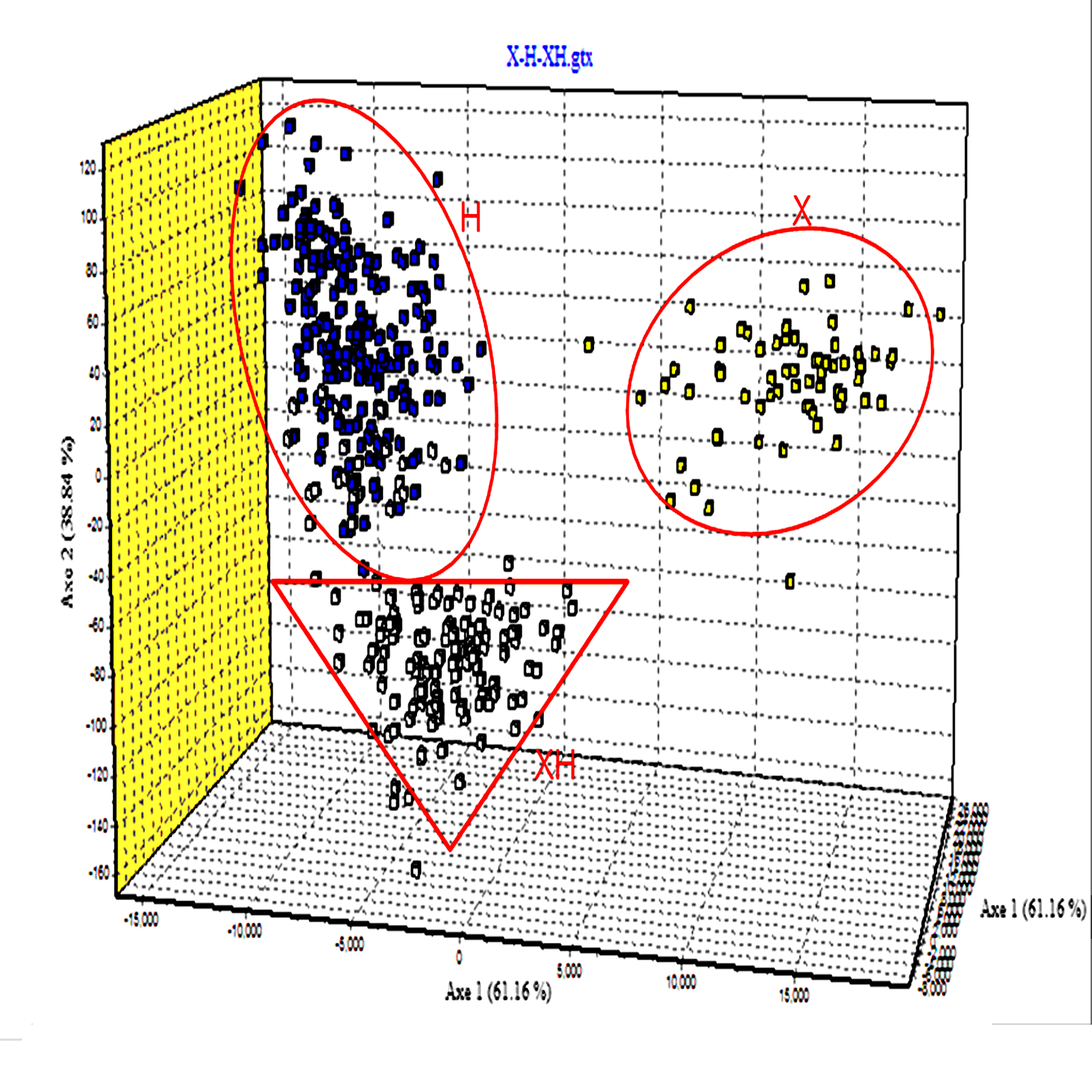

Supplement: Additional file 11: Figure S8 — Factorial Correspondence Analysis (FCA) based on nine microsatellite loci in A. baerii, H. Dauricus and their hybrids. The figure shows the distinction between A. baerii, H. dauricus and A. baerii♀ × H. dauricus♂. [file 1297-9686-45-21-S11.png]

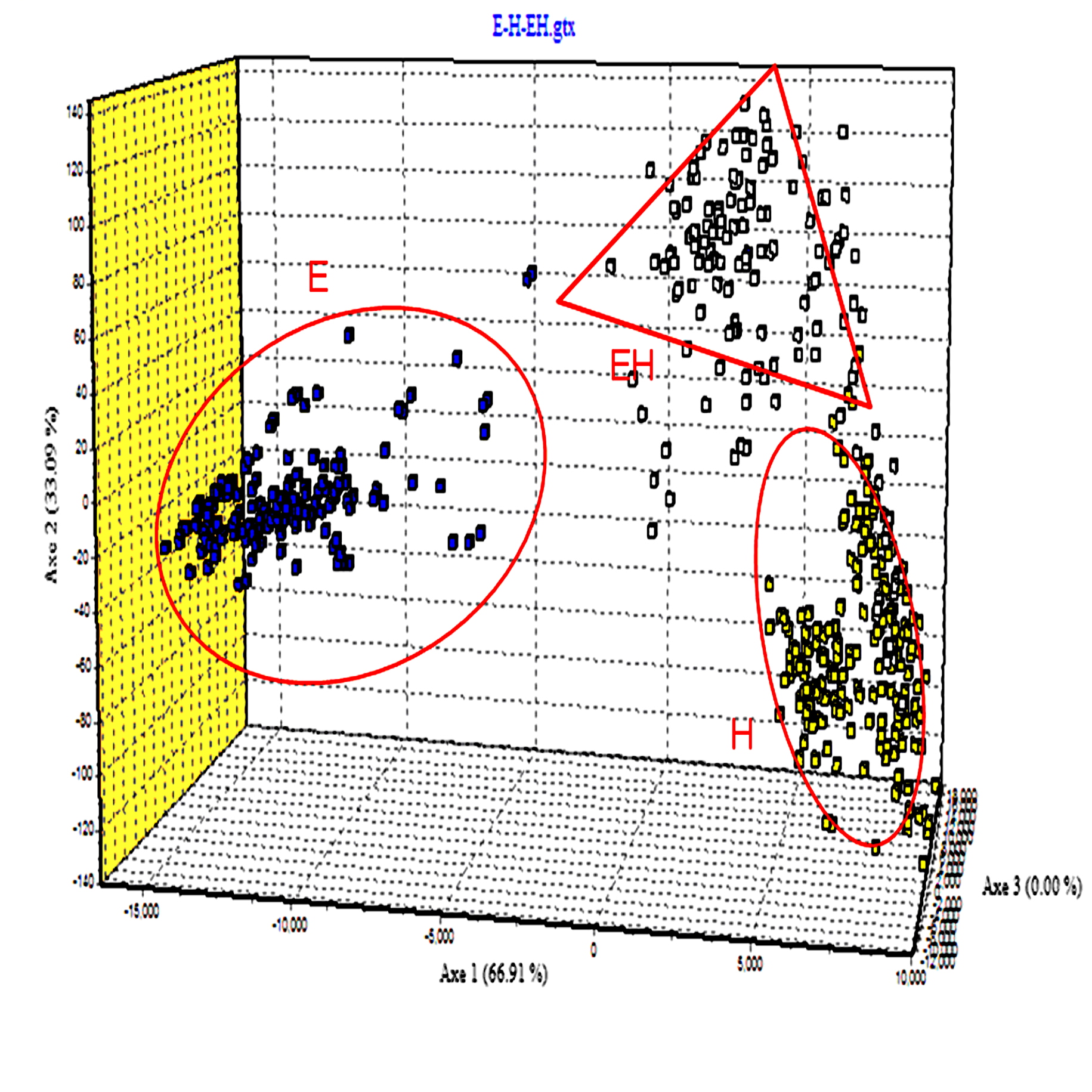

Supplement: Additional file 12: Figure S9 — Factorial Correspondence Analysis (FCA) based on nine microsatellite loci in A. gueldenstaedti, H. dauricus and their hybrids. The figure shows the distinction between A. gueldenstaedti, H. dauricus and A. gueldenstaedti♀ × H. dauricus♂. [file 1297-9686-45-21-S12.png]

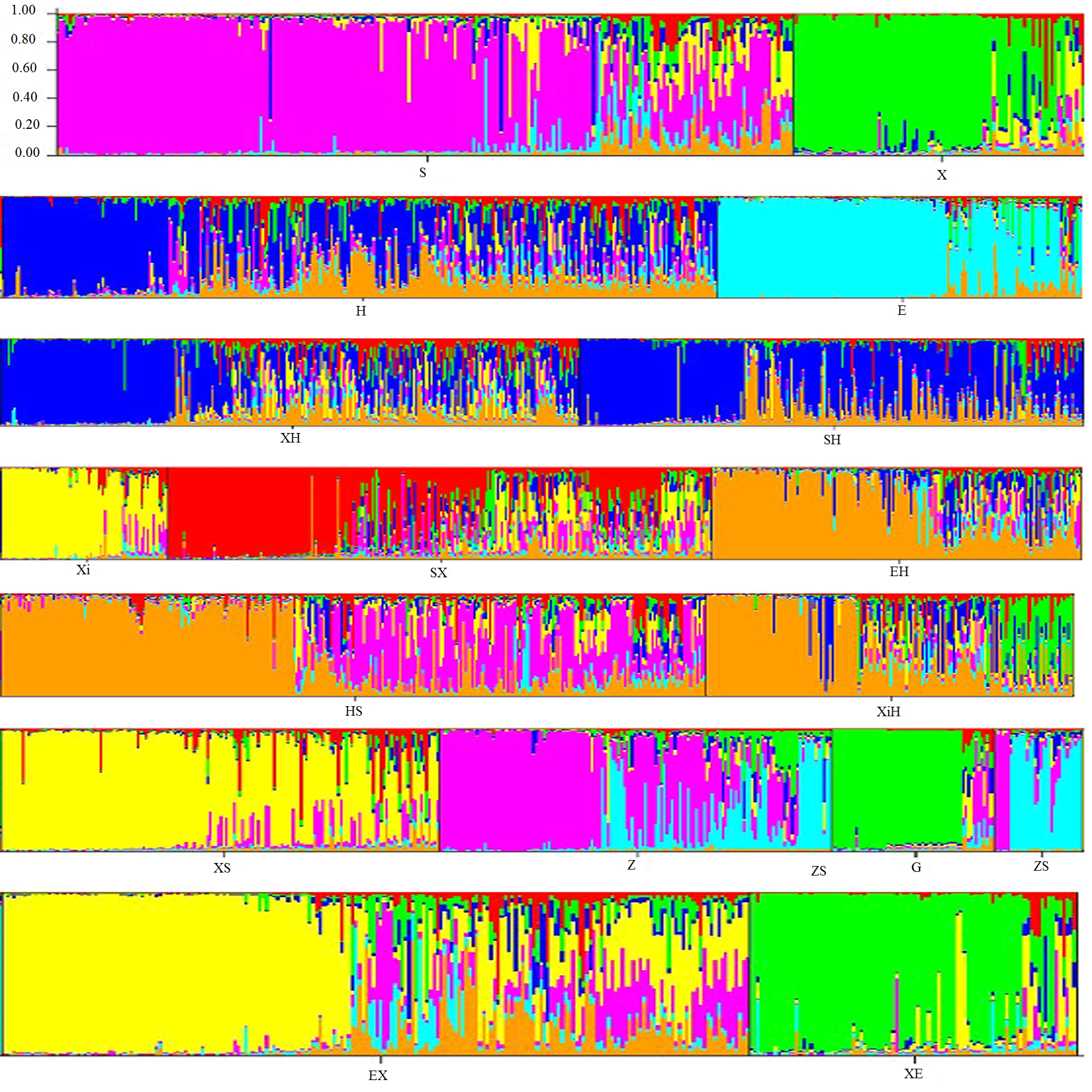

Supplement: Additional file 13: Figure S3 — Assignation of the 466 sturgeons by STRUCTURCE analysis based on nine microsatellite loci in 17 sturgeon strains. The figure illustrates the existence of different genetic clusters in sturgeon breeds revealed the by the analysis of microsatellite genotyping data. [file 1297-9686-45-21-S13.png]
