# Supplementary material for: Genetic variation and relationships of seven sturgeon species and ten interspecific hybrids
Source: Genet Sel Evol. 2013 Jun 28;45(1):21. doi: 10.1186/1297-9686-45-21 (PMC3704922; doi:10.1186/1297-9686-45-21)
Supplement: Additional file 4: Table S3 — Total number of alleles detected for the nine microsatellite loci selected for the analysis of 17 sturgeon strains. The data provides the number of alleles detected for the nine microsatellite loci by POLYSAT. [file 1297-9686-45-21-S4.doc]

| Species Locus | AS100 | HLJSX24 | LS19 | LS54 | LS68 | SPL113 | SPL120 | SPL168 | SPL106 | Total |
| --- | --- | --- | --- | --- | --- | --- | --- | --- | --- | --- |
| *A. schrenckii* | 12 | 10 | 9 | 7 | 15 | 11 | 13 | 15 | 7 | 99 |
| *A. baerii* | 13 | 17 | 6 | 8 | 15 | 9 | 9 | 7 | 10 | 94 |
| *H. Dauricus* | 13 | 10 | 4 | 4 | 12 | 5 | 9 | 9 | 8 | 74 |
| *A. gueldenstaedti* | 11 | 11 | 11 | 11 | 11 | 8 | 11 | 12 | 3 | 89 |
| *A. ruthenus* | 14 | 9 | 8 | 7 | 13 | 8 | 8 | 10 | 12 | 89 |
| *A. sinensis* | 16 | 15 | 4 | 7 | 10 | 11 | 7 | 18 | 9 | 97 |
| *A. stellatus* | 6 | 6 | 6 | 6 | 5 | 4 | 4 | 0 | 6 | 43 |
| *A. baerii*♀×*H. Dauricus*♂ | 7 | 6 | 7 | 5 | 9 | 5 | 4 | 6 | 11 | 60 |
| *A. schrenckii*♀×*A. baerii*♂ | 12 | 10 | 8 | 8 | 15 | 9 | 11 | 12 | 8 | 93 |
| *A. gueldenstaedti*♀×*H. Dauricus*♂ | 11 | 9 | 10 | 9 | 12 | 8 | 9 | 13 | 9 | 90 |
| *H. Dauricus*♀×*A. schrenckii*♂ | 20 | 16 | 4 | 8 | 15 | 11 | 11 | 18 | 14 | 117 |
| *A. ruthenus*♀×*H. Dauricus*♂ | 11 | 6 | 11 | 6 | 17 | 5 | 5 | 8 | 13 | 82 |
| *A. baerii*♀×*A. schrenckii*♂ | 12 | 7 | 6 | 6 | 9 | 6 | 8 | 5 | 6 | 65 |
| *A. baerii*♀×*A. gueldenstaedti*♂ | 8 | 3 | 8 | 11 | 13 | 8 | 9 | 10 | 6 | 76 |
| *A. gueldenstaedti*♀×*A.baerii*♂ | 9 | 16 | 10 | 13 | 13 | 10 | 9 | 13 | 8 | 101 |
| *A. schrenckii*♀×*H. Dauricus*♂ | 11 | 14 | 4 | 4 | 14 | 7 | 6 | 9 | 8 | 77 |
| *A. sinensis*♀×*A. schrenckii*♂ | 4 | 8 | 3 | 3 | 5 | 2 | 3 | 10 | 4 | 42 |
| overall | 54 | 27 | 30 | 25 | 49 | 45 | 26 | 59 | 71 | 386 |

Table S3 Total number of alleles detected in nine microsatellite loci selected for 17 sturgeon strains
