# Supplementary material for: Genetic variation and relationships of seven sturgeon species and ten interspecific hybrids
Source: Genet Sel Evol. 2013 Jun 28;45(1):21. doi: 10.1186/1297-9686-45-21 (PMC3704922; doi:10.1186/1297-9686-45-21)
Supplement: Additional file 5: Table S4 — Average number of alleles per individual per locus detected in 17 sturgeons. The data provides the average number of alleles per individual per locus detected in 17 sturgeon strains. N is the total number of individuals for each sturgeon strain. [file 1297-9686-45-21-S5.doc]

Table S4 Average number of alleles per individual per locus detected in 17 sturgeon strains

| Population | N | AS100 | HLJSX24 | LS19 | LS54 | LS68 | SPL113 | SPL120 | SPL168 | SPL106 |
| --- | --- | --- | --- | --- | --- | --- | --- | --- | --- | --- |
| *A. schrenckii* | 30 | 0.4 | 0.33 | 0.3 | 0.23 | 0.5 | 0.37 | 0.43 | 0.5 | 0.23 |
| *A. baerii* | 30 | 0.43 | 0.57 | 0.2 | 0.27 | 0.5 | 0.3 | 0.3 | 0.23 | 0.33 |
| *H. Dauricus* | 26 | 0.5 | 0.38 | 0.15 | 0.15 | 0.46 | 0.19 | 0.35 | 0.35 | 0.31 |
| *A. gueldenstaedti* | 34 | 0.32 | 0.32 | 0.32 | 0.32 | 0.32 | 0.24 | 0.32 | 0.35 | 0.09 |
| *A. ruthenus* | 23 | 0.61 | 0.39 | 0.35 | 0.3 | 0.57 | 0.35 | 0.352 | 0.43 | 0.52 |
| *A. sinensis* | 25 | 0.64 | 0.6 | 0.16 | 0.28 | 0.4 | 0.44 | 0.28 | 0.72 | 0.36 |
| *A. stellatus* | 21 | 0.29 | 0.29 | 0.29 | 0.29 | 0.24 | 0.19 | 0.19 | 0 | 0.29 |
| *A. baerii*♀×*H. Dauricus*♂ | 31 | 0.23 | 0.19 | 0.23 | 0.16 | 0.29 | 0.16 | 0.13 | 0.19 | 0.35 |
| *A. gueldenstaedti*♀×*A. baerii*♂ | 40 | 0.23 | 0.4 | 0.25 | 0.33 | 0.33 | 0.25 | 0.23 | 0.33 | 0.2 |
| *A. schrenckii*♀×*H. Dauricus*♂ | 26 | 0.42 | 0.54 | 0.15 | 0.15 | 0.54 | 0.27 | 0.23 | 0.35 | 0.31 |
| *A. schrenckii*♀×*A. baerii*♂ | 30 | 0.4 | 0.33 | 0.27 | 0.27 | 0.5 | 0.3 | 0.37 | 0.4 | 0.27 |
| *A. gueldenstaedti*♀×*H. Dauricus*♂ | 30 | 0.37 | 0.3 | 0.33 | 0.3 | 0.4 | 0.27 | 0.3 | 0.43 | 0.3 |
| *H. Dauricus*♀×*A. schrenckii*♂ | 30 | 0.67 | 0.53 | 0.13 | 0.27 | 0.5 | 0.367 | 0.37 | 0.6 | 0.47 |
| *A. ruthenus*♀×*H. Dauricus*♂ | 30 | 0.37 | 0.2 | 0.37 | 0.2 | 0.57 | 0.17 | 0.17 | 0.27 | 0.43 |
| *A. baerii*♀×*A. schrenckii*♂ | 32 | 0.38 | 0.22 | 0.19 | 0.19 | 0.28 | 0.19 | 0.25 | 0.16 | 0.19 |
| *A. baerii*♀×*A. gueldenstaedti*♂ | 23 | 0.35 | 0.13 | 0.35 | 0.48 | 0.57 | 0.35 | 0.39 | 0.43 | 0.26 |
| *A. sinensis*♀×*A. schrenckii*♂ | 5 | 0.8 | 1.6 | 0.6 | 0.6 | 1 | 0.4 | 0.6 | 2 | 0.8 |

N is the total number of individuals at each sturgeon
